# Effects of Protein-Rich Nutritional Composition Supplementation on Sarcopenia Indices and Physical Activity during Resistance Exercise Training in Older Women with Knee Osteoarthritis

**DOI:** 10.3390/nu13082487

**Published:** 2021-07-21

**Authors:** Chun-De Liao, Yi-Hung Liao, Tsan-Hon Liou, Ching-Ya Hsieh, Yu-Chi Kuo, Hung-Chou Chen

**Affiliations:** 1 Master Program in Long-Term Care, College of Nursing, Taipei Medical University, Taipei 110301, Taiwan; 08415@s.tmu.edu.tw; 2 Department of Physical Medicine and Rehabilitation, Shuang Ho Hospital, Taipei Medical University, New Taipei City 235041, Taiwan; peter_liou@s.tmu.edu.tw; 3 Department of Exercise and Health Science, National Taipei University of Nursing and Health Sciences, Taipei 112303, Taiwan; yihungliao@gmail.com (Y.-H.L.); amyhsieh.sky@gmail.com (C.-Y.H.); yuchi@ntunhs.edu.tw (Y.-C.K.); 4 Department of Physical Medicine and Rehabilitation, School of Medicine, College of Medicine, Taipei Medical University, Taipei 110301, Taiwan; 5 College of Human Development and Health, National Taipei University of Nursing and Health Sciences, Taipei 112303, Taiwan

**Keywords:** sarcopenia, osteoarthritis, protein supplementation, resistance training, lean mass, physical activity

## Abstract

Older adults with knee osteoarthritis (KOA) are at high risk of sarcopenia. Protein-rich nutritional composition supplementation (PS) combined with resistance exercise training (RET) improves muscle gains and facilitates physical activity in older adults. However, whether PS augments the effects of RET on muscle mass and PA in patients with KOA remains unclear. Therefore, this study identified the effects of PS on sarcopenic indices and PA in older women with KOA subjected to an RET program. Eligible older women aged 60–85 years and diagnosed as having KOA were randomly assigned to either the experimental group (EG) or the control group (CG). Both groups performed RET twice a week for 12 weeks. The EG received additional PS during this period. Outcome measures included appendicular lean mass index, walking speed, physical activity, and scores on the Western Ontario and McMaster Universities Osteoarthritis Index—WOMAC). All measures were tested at baseline and after intervention. With participant characteristics and baseline scores as covariates, analysis of variance was performed to identify between-group differences in changes in all outcome measures after intervention. Statistical significance was defined as *p* < 0.05. Compared with the CG, the EG achieved greater changes in appendicular lean mass index (adjusted mean difference (aMD) = 0.19 kg/m^2^, *p* < 0.01), physical activity (aMD = 30.0 MET-hour/week, *p* < 0.001), walking speed (aMD = 0.09 m/s, *p* < 0.05), and WOMAC global function (aMD = −8.21, *p* < 0.001) after intervention. In conclusion, PS exerted augmentative effects on sarcopenic indices, physical activity, and perceived global WOMAC score in older women with KOA through 12 weeks of RET.

## 1. Introduction

Knee osteoarthritis (KOA) is one of the most prevalent musculoskeletal diseases in older adults [1], with negative effects on both individuals’ physical well-being and public health [2]. Patients with KOA experience pain, impaired physical mobility, and limitations in functional activities [3,4], which are associated with physical inactivity [5] and ultimately lead to disability [6].

Pain and muscle weakness are the main clinical features of KOA [7,8]. Muscle weakness in KOA has been linked to sarcopenia [9,10,11,12,13,14,15,16], a condition characterized by age-related muscle attenuation in older adults [17]. Several observational studies have indicated that patients with KOA have a significantly lower percentage of lean body mass than their counterparts without this condition [16,18,19]. Lee et al. observed that a low skeletal muscle mass index value in the legs is an independent risk factor for KOA [20]; furthermore, Kim et al. indicated that the skeletal muscle mass index is significantly negatively associated with the Kellgren-Lawrence classification for KOA [10]. One recent study reported that older adults with KOA are at high risk of sarcopenia [21]. Given that lean leg mass is closely associated with muscle power in KOA [22] and that low levels of skeletal muscle mass are closely associated with physical difficulties and poor health status in elderly patients [23,24], sarcopenia may be independently associated with muscle weakness and physical decline over the course of KOA, which further highlights the role of muscle in the genesis and management of KOA [25,26]. Therefore, the maintenance of muscle strength and the prevention of sarcopenia are critical for enabling older adults with KOA to perform activities of daily living independently.

Aging-related loss of skeletal muscle mass is more advanced in frail older adults, especially in those with chronic diseases, such as osteoarthritis or neurological disorders [17]. In addition, such muscle attenuation is primarily characterized by type II myofiber atrophy and has been found to manifest as smaller muscle fiber size as opposed to fiber loss [27,28]. Studies have identified that resistance exercise training (RET) effectively mitigates sarcopenia in terms of satellite cell proliferation and increased synthesis rates of muscle contractile and mitochondrial proteins, which further contribute to myofiber hypertrophy [28,29]. Among the various types of exercise, RET with elastic bands or tubes has been frequently employed as a safe muscle strengthening intervention for older adults [30,31]. In one study, individuals engaging in elastic and conventional free-weight RET exhibited similar muscle activation patterns and comparable self-perceived efficacy [32]. RET with elastic bands can be conveniently performed at home and strengthens the musculoskeletal system, further enhancing physical mobility [33,34]. In addition, studies have supported the benefits for sarcopenia prevention of RET with elastic bands or tubes [35,36]; this method is in line with the recommended clinical practice guidelines for sarcopenia [37].

KOA is recognized as a serious musculoskeletal disease [38]. The management of mild-to-moderate KOA has included multidisciplinary interventions, such as the combination of pain medication with nonpharmacological treatments [39]. Exercise training constitutes a first-line treatment for KOA [40] and has been effectively employed to increase muscle mass, strength, and physical function [41]. Additionally, dietary interventions such as dietary protein or protein supplementation (PS) have been incorporated into multidisciplinary management for KOA [42,43,44]. In light of statistics indicating that 30.3–65.1% of older adults with KOA fail to meet the protein recommended dietary allowance (RDA) of 1.2–1.5 g/kg/day for older adults who have chronic diseases [45]. PS and protein-based diet interventions are believed to augment the efficacy of exercise training in older adults [46,47]. A multitude of previous studies demonstrate consistent results indicating that PS plus RET (PS + RET) obtains greater muscle mass gain and function restoration compared to RET alone [48,49,50,51,52]. However, most of the previous trials enrolled sarcopenic or frail older adults and few focused on the elder people with KOA. Therefore, it remains unclear whether PS augment any additional effect of RET on sarcopenia-related outcomes for KOA population. Because older adults with KOA face a high risk of sarcopenia and that condition in turn may affect their functional outcomes, skeletal muscle has been targeted for KOA management [25,26]. Identifying the efficiency and efficacy of PS + RET is crucial for preserving muscle mass in older adults with KOA. Therefore, the present study aimed to examine the effects of PS + RET on sarcopenia index, physical activity, and global functional outcomes in older adults with KOA.

## 2. Materials and Methods

### 2.1. Study Design

This prospective randomized controlled trial had two parallel study arms. The study protocol was executed at the rehabilitation center of a university-based hospital. This study was registered in the Chinese Clinical Trial Registry (registry number ChiCTR-IPR-17012106) and received ethical approval from the Joint Institutional Review Board of Taipei Medical University (trial number N201804049). Participants were enrolled from August 2018 to March 2020. All the participants provided consent at baseline admission 2 weeks prior to the intervention. At baseline, each patient was randomized into one of two groups: an experimental group (EG) receiving elastic PS + RET and an age-matched control group (CG) receiving RET alone. After enrollment, data on the participants′ demographic characteristics and comorbidities were extracted from a standard medical chart review, and a comorbidity score for each patient was calculated using the cumulative illness rating scale [53]. A 12-week intervention was employed for all participants in the CG and EG, which was followed by a follow-up period of 6 months. During the postintervention follow-up period, all participants were instructed to maintain individual dietary habits and regular physical activities, and a research assistant confirmed that all participants followed the instructions through monthly phone contact. The outcome data were collected by two trained research assistants who were blinded to the group assignment at baseline (i.e., pretest before intervention) and posttest after 12 weeks of intervention.

### 2.2. Participants

Female patients who were aged ≥60 years and had primary KOA, diagnosed according to the criteria of the American College of Rheumatology [54], were recruited from the outpatient clinic of the Department of Orthopedics at our hospital. All participants had knee pain from the affected joint, both at rest and during activity in daily life, and typical clinical features and radiographic evidence (Kellgren-Lawrence grade I–III) of KOA. The participants were excluded if they had any of the following conditions: (a) history of hip or knee arthroplasty; (b) sensitivity or allergy to milk proteins or impaired renal function; (c) uncontrolled hypertension; (d) any cardiovascular or pulmonary disease that would impede them from engaging in an exercise study; or (e) neurological or cognitive impairment.

In the present study, the sex-specific design was conducted for older female participants based on the following reasons. First, sex-specific adaptations in response to RET have been identified for older people KOA [55]. In addition, the criteria for the classification of sarcopenia status differ between men and women in elderly [17,56] and KOA [57] populations. Moreover, older female patients have significantly lower muscle mass [58] and strength [59] than their male peers. Therefore, pooling the measured data of the two sexes into one group to analyze sarcopenia indices is difficult, and we considered a sex-specific study design to reduce such biases caused by sex differences in the analysis of intervention outcomes.

### 2.3. Sample Size Estimation

The sample size was estimated based on a lean mass difference of 0.74 kg between the PS + RET group and the RET group [47]. With a statistical power of 0.8 and an alpha value of 0.05, we determined that a minimum of 58 participants (29 per group) would be required to identify a significant between-group difference (assuming a standard deviation of 0.83) in lean mass gain [60]. We set the recruitment target as 72 participants (36 for each group) to allow for a maximum of 25% of enrolled participants to withdraw before the end of the study.

### 2.4. Randomization

Group allocation at baseline was random, involving the distribution of sealed opaque envelopes. A list of computer-generated random numbers provided by an independent randomization center was used in stratified permuted blocks (size 4). Concealed assignment was performed independently for both groups by an independent principal investigator who was not involved in the enrollment, intervention, or assessment.

### 2.5. Elastic Resistance Exercise

A home-based RET regime conducted in accordance with our earlier exercise protocol [36] was employed using Thera-Band products (Hygenic Co., Akron, OH, USA), the colors (yellow, red, green, blue, black, and silver) of which denote the degree of elasticity and the corresponding resistance level. Details on the exercise regime and exercise progression protocol are presented in Tables S1 and S2, respectively. To standardize the tension (i.e., elastic force) across participants for all training bands and movements, the individual distance from the upper and lower limb to the fixed point on the chair (or wall) was determined respectively at the onset of the trial. For each movement, the individual distance was judged to enable the participants to stretch a 100-cm band to an elongation of 150% of the resting length (i.e., 150 cm) throughout the full range of the movement. Therefore, the distance between the body part and fixed place was unique for each participant and the tension of each band remained constant across all participants for all exercise movements. When the bands were stretched 150% of the resting length, loading weights corresponded to 0.83 kg, 1.10 kg, 1.24 kg, 1.70 kg, 2.18 kg, and 2.58 kg for the yellow, red, green, blue, black, and silver bands, respectively [61]. All participants were provided with an individualized exercise book containing instructions and images of exercise movements.

The elastic RET program was performed on 2 nonconsecutive days weekly. The initial 2 weeks of training sessions were a familiarization period, the aim of which was to ensure that each movement or exercise was performed equally precisely at home and at the clinic. During the familiarization period, all training sessions were supervised by a licensed senior physical therapist who was unaware of the study group assignment and were conducted for all participants in the CG and EG to ensure that standardized postures were maintained in self-administered training sessions. The training session was then continued twice a week (i.e., two nonconsecutive days per week) during the home exercise period and all participants in both groups were instructed not to perform the elastic RET on the other weekdays (Table S1). To control the variation in subjective efforts with the physical intensity of individual rehabilitation protocols, a Borg Rating of Perceived Exertion (RPE) scale was used to rate the patients’ perceived exertion during the training sessions to control the variation in subjective efforts with the physical intensity of individual rehabilitation protocols [32,62]. At each training session, an exercise intensity with a perception of effort ranging from moderate to high level was prescribed for all participants according to the guidelines of the American College of Sports Medicine [63] (Table S2). For each resistance grade, each participant was instructed to yield an RPE level of “somewhat hard (moderate)” but not exceed the “hard (high)” level, corresponded with ratings of 13 to 15 on the Borg RPE scale [62]. In addition, a Borg RPE rating of 13–15 is equivalent to a score of 6 (i.e., somewhat hard) to 8 (i.e., hard) on the 10-point Omni Perceived Exertion Scale-Resistance Exercise Scale (OMNI-RES) [64,65] despite the fact that OMNI-RES has been validated to monitor the exercise intensity of elastic RET [66] and can be used to identify training-induced strength changes in older adults [67]. The training loads increased when the perceived difficulty falls below 13 on the Borg RPE scale. Each exercise session consisted of a 10-min warm-up, upper and lower quarter movements, and a 10-min cool-down (Table S2). Exercise movements were designed based on elastic exercise regimes previously established for training older adults [68,69,70,71]. The participants progressed from yellow to silver bands over the 12-week intervention period. Three sets, involving 10 to 20 repetitions of gentle concentric and eccentric contractions (3 s duration per each contraction) through the full range of the motion and a rest interval of 30 s between sets, were performed for each action. The exercise intensity was increased through band upgrades (e.g., yellow-red progression) when the participants met their targets for perceived exertion. If the participants could not tolerate successive advancements in exercise load, they continued using bands of the same color, performing an additional two to three sets of each exercise until the required effort could be exerted. An exercise logbook was given to each participant to record their progress over the intervention period. During the home exercise period, the physical therapist monitored patient compliance with the exercise regimen and adherence with progression by weekly phone contact.

### 2.6. Dietary Protein Supplementation

The nutritional supplements (AFFIX HEALTH PTE. Ltd., Singapore, Taiwan Branch) were in powder form and dissolved in 200 mL of water before consumption. Each 24 g serving contained 66% of protein (whey 7.0 g, leucine 2.2 g), 28% of carbohydrates, and 6% of fat, corresponding to a total caloric content of 85 kcal per serving (Table S3). All participants in the EG were asked to consume the protein-enriched supplement twice a day (one serving at breakfast and lunch, respectively, on the non-exercise day). On the training day, participants were instructed to consume two servings (one at breakfast and one within 30 min of RET) of protein-enriched supplements during the intervention period. The participants were instructed to consume the same dose of supplements on the scheduled training days even if the RET was not performed due to any cause. Through weekly phone contact, a research dietician monitored participant compliance with the supplement regimen.

### 2.7. Dietary Intake

A 3-day food diary was used to assess potential changes in habitual daily food intake during the intervention period. All participants were instructed to maintain their regular dietary habits and write in their food diary before and after intervention [72]. Food intake was recorded on 2 and 1 days during the week and weekend, respectively. At baseline admission, the participants were instructed on how to complete the dietary records. Dietary intake data were analyzed using the open-source software MyFitnessPal (MyFitnessPal, Inc., San Francisco, CA, USA) [73], which has been employed to analyze food intake data in previous studies [74,75]. The nutritional supplements that were consumed on training days were not included in the dietary analysis.

### 2.8. Primary Outcome Measures

The primary outcomes of interest in this study were sarcopenic indexes, which included muscle mass, walking speed, and handgrip strength, which have been used as indicators of sarcopenia [17,56]. Muscle mass was determined through bioelectrical impedance analysis (InBody 770, Upwards Biosystems, Ltd., Taipei, Taiwan) by an investigator blinded to the group allocation. The analytical output included whole-body skeletal muscle mass and appendicular lean mass. Following the recommendations of the Asian Working Group for Sarcopenia and the European Working Group on Sarcopenia in Older adults, two relative indexes, the appendicular lean mass index (AMI, kg/m^2^) and whole-body skeletal muscle mass index (SMI, kg/m^2^), were estimated to define muscle mass outcomes [17,56]. The sarcopenic indexes, AMI and SMI, were calculated as the appendicular lean mass and whole-body skeletal muscle mass divided by squared height in meters, respectively.

Walking speed was determined by the 10 m walk test measuring the time required for the patient to travel 10 m on a track at a self-determined pace. The use of a walking aid was allowed depending on the participants’ tolerance of knee pain during the walk test.

Muscle quality (MQ) is defined as an index of muscle strength or power relative to muscle mass [76]. One study reported that the muscle quality index more accurately predicted low physical function in older women than absolute muscle strength [77]. The MQ index was estimated based on handgrip strength (kg) divided by lean arm mass (kg). Static handgrip strength was measured using a handheld dynamometer (JAMAR Hydraulic Dynamometer, Kam Wah Enterprises Co., Taipei, Taiwan) [78].

### 2.9. Secondary Outcome Measures

Physical activity was assessed using International Physical Activity Questionnaire Short Form (IPAQ-SF). The IPAQ-SF comprises seven open-ended questions concerning individuals’ recollection of their physical activity during the last 7 days [79]. One study confirmed the validity and reliability of the IPAQ-SF for older adults with osteoarthritis [80]. Each type of activity was transformed into a metabolic equivalent (MET) score, which was defined elsewhere [81]. All data were expressed as MET-hour per week.

The self-reported function was assessed using the Western Ontario and McMaster Universities Osteoarthritis Index (WOMAC), which was developed specifically for KOA [82]. Specifically, the Chinese version of the WOMAC questionnaire was employed. It has favorable validity and reliability, with an intraclass correlation coefficient that ranges from 0.80 to 0.98 [83]. The 24-item questionnaire consists of three domains assessing pain, stiffness, and physical difficulty in activities of daily living. The global (i.e., total) score was standardized to a range of 0 to 100, with higher scores representing poorer health.

### 2.10. Statistical Analysis

Independent *t*-tests and chi-squared analysis were conducted to examine between-group differences at baseline in the demographic characteristics, including age, height, weight, body mass index (BMI), comorbidity scores [53], and the outcome measures. The Kolmogorov-Smirnov test, performed for all variables, confirmed that the data followed a Gaussian distribution. All analyses were conducted on an intention-to-treat analysis, using the last observation carried forward method to impute any missing data [84]. One-way analysis of covariance was performed to assess between-group differences in changes at posttest from pretest, respectively, using the participants’ characteristics and the baseline scores at pretest as the covariates. In addition, post hoc analysis was performed using the Bonferroni method.

At each time point, the participants were classified as having presarcopenia, sarcopenia, or severe sarcopenia on the basis of the following established outcome measure cutoffs for older Asian women [56]: AMI of <5.7 kg/m^2^ for low muscle mass, walking speed of <1.0 m/s for low physical performance, and handgrip strength of <18 kg for low muscle strength. Participants with low muscle strength alone, low physical performance alone, or with both but without low muscle mass were classified as having presarcopenia (or possible sarcopenia). Sarcopenia was defined as having low muscle mass in combination with either low muscle strength or low physical performance, and severe sarcopenia was defined as having low muscle mass, low muscle strength, and low physical performance [56]. Proportions of each classification for sarcopenic status within each group were calculated. Chi-squared analyses were performed to examine the effects of PS + RET on sarcopenia.

SPSS Statistics for Windows, Version 22.0 (IBM Corp., Armonk, NY, USA) was used for all analyses. Differences were considered significant at *p* < 0.05.

## 3. Results

### 3.1. Patient Demographics and Clinical Characteristics

**Figure 1:**
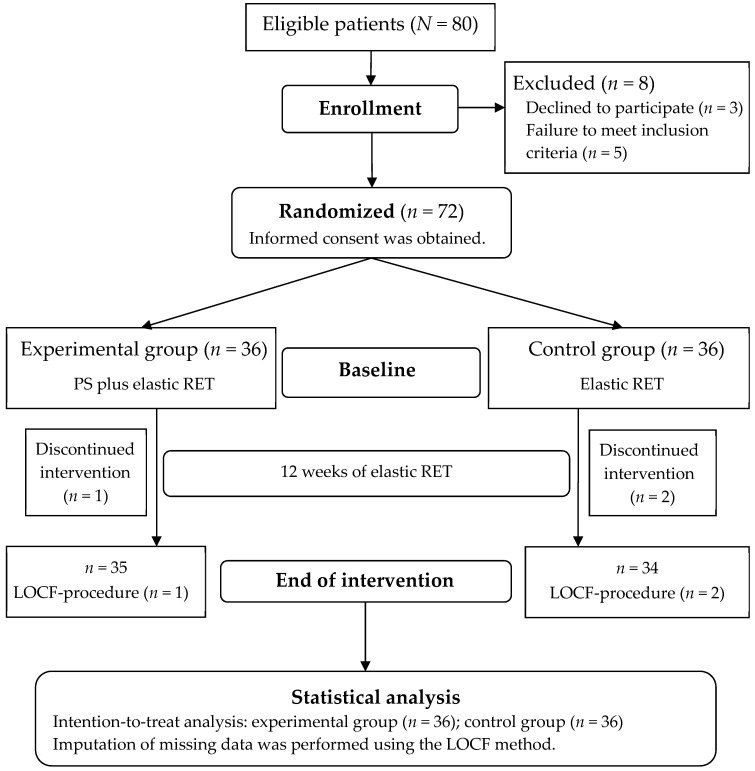
Study flowchart. LOCF, last observation carried forward; PS, protein supplement; RET, resistance exercise training.

The Consolidated Standards of Reporting Trials flow diagram of patient enrollment and allocation is presented in Figure 1. The sample comprised 72 older women, whose mean (range) age, mean (range) BMI, and mean (range) disease duration was 69.2 (56–85) years, 27.8 (19.8–36.1) kg/m^2^, and a mean disease duration of 9.6 (0.4–37.8) years. All included participants were randomly allocated to the EG (*n* = 36) or CG (*n* = 36) after they provided informed consent at pretest. Overall, 35 and 34 participants in the EG and CG completed the 3-month follow-up assessment at posttest. On the basis of the intention-to-treat principle, the missing data were imputed through the last observation carried forward method.

The patient demographics and clinical characteristics are presented in Table 1. No significant between-group differences in characteristics were observed (all *p* > 0.05). A total of 29 participants (13 in the CG, 16 in the EG) had walking limitations and walked using a walking aid. All participants reported knee pain with a mean (SD) WOMAC pain score of 11.6 (2.6). In total, 63 participants (30 in the CG, 33 in the EG) had mild KOA with a Kellgren-Lawrence grade ≤II whereas 9 participants (6 in the CG, 3 in the EG) experienced moderate KOA (Kellgren-Lawrence grade III).

Regarding sarcopenia status of the participants, 65 (90.3%), 34 (47.2%), and 26 (36.1%) out of 72 participants experienced low physical performance (i.e., slow walking speed <1.0 m/s), low muscle strength (i.e., handgrip strength <18 kg), and low muscle mass (i.e., AMI < 5.7 kg/m^2^) at baseline. Accordingly, a total of 69 (95.8%) participants with either slow walking speed or low handgrip strength were classified as having potential sarcopenia risk, among which 26 (36.1%) participants with low muscle mass were finally classified as having sarcopenia or severe sarcopenia.

### 3.2. Dietary Intake and Energy Expenditure during the Study Period

Analytical results for the 3-day food diary (excluding supplement intake) revealed no significant differences between the PS + RET and RET groups at pretest (Table 2). The mean dietary protein intake was 0.98 g/kg/day (standard deviation = 0.44) for the total sample, with 48.6% of participants (16 in the CG; 19 in the EG) having an insufficient protein intake of less than 0.8 g/kg/day at pretest. All the diet intake measures and daily energy expenditure at pretest did not differ between the PS + RET and RET groups (*p* > 0.05).

During RET intervention period, daily protein intake from diet (excluding PS) did not differ between the two groups (*p* > 0.05) as well as daily total energy intake did (*p* > 0.05; Table 2). Taking into account the PS, total protein intake of EG increased to 1.21 ± 0.34 g/kg/day during the 12-week intervention period. Throughout the study period, habitual daily total energy intake increased over time (*p* < 0.05) but did not differ between groups (*p* > 0.05). The percentage contributions from macronutrient composition did not change over time or differ between groups (*p* > 0.05; Table 2).

### 3.3. Primary and Secondary Outcomes

The effects of PS + RET on changes in sarcopenia indices, physical activity, and WOMAC outcomes at posttest are presented in Table 3. For details on the time scores for all outcome measures, please see Table S4.

#### 3.3.1. Effects on Sarcopenic Indices

After adjustment for baseline scores and participant characteristics (i.e., age, comorbidity scores, disease severity, and disease duration), the PS + RET intervention was associated with greater increases in SMI (aMD = 0.21 kg, *p* < 0.05) and AMI (aMD = 0.19 kg, *p* < 0.01) at posttest compared with those from RET alone (Table 3). At posttest (Table 3), participants in the EG had faster walking speed (aMD = 0.09 m/s, *p* < 0.05) compared with peers in the CG. No significant between-group differences were noted in changes of MQ at posttest (*p* > 0.05).

#### 3.3.2. Effects on Physical Activity and Global Function

After 12 weeks of PS + RET, with adjustment for baseline data and participant characteristics, the EG exhibited significantly greater changes in total physical activity (aMD = 30.0 MET-hour/week, *p* < 0.001). Similar results were observed in vigorous (aMD = 4.98 MET-hour/week, *p* < 0.05), moderate (aMD = 3.41 MET-hour/week, *p* < 0.05), and light (aMD = 18.4 MET-hour/week, *p* < 0.001) physical activity.

Compared with the CG, the EG reported greater mitigation in pain on the WOMAC pain (aMD = −1.37, *p* < 0.05) and alleviation of physical difficulty (aMD = −6.49, *p* < 0.001), as well as greater improvements in global function (aMD = −8.21, *p* < 0.001) at posttest when age, comorbidity scores, disease severity, disease duration, and baseline scores were controlled for (Table 3).

### 3.4. Effects on Sarcopenia

The chi-squared test revealed that PS + RET exerted effects on sarcopenia prevention in the EG (Table 4), in which the number of participants without sarcopenia increased significantly over time (*p* < 0.001), and this was also the case in the CG (*p* = 0.004). In addition, the EG had a greater number of participants who yielded nonsarcopenia than the CG did at the posttest (22 *versus* 10; *p* = 0.004). Moreover, the number of participants classified as having severe sarcopenia was significantly lower in the EG than in the CG (one *versus* six; *p* < 0.05) at posttest.

### 3.5. Compliance with Protein Supplementation and Exercise

On average, compliance with the RET program in the CG and EG was 82.6% and 84.3% of the expected exercise sessions, respectively. Adherence for each resistance level was presented in Table S5. All participants in each group successfully yielded yellow, red, and green Thera-Band. The number of participants able to tolerate the highest resistance (i.e., the silver Thera-Band) in the CG and EG (26 and 28 individuals, respectively) did not differ significantly (*p* > 0.05). Based on close online monitoring, all members of the EG were confirmed to have completed their supplementation regimens, corresponding to a 100% adherence rate. No participant discontinued supplementation because of discomfort or gastrointestinal complaints. No serious side effects were reported during the intervention and follow-up periods.

## 4. Discussion

### 4.1. Summary of the Main Findings

In this study, we investigated the effects of PS on sarcopenic indices, physical activity, and global function during a 12-week RET intervention for older women with KOA. The main findings are as follows: First, the EG (PS + RET) exhibited significantly greater improvements in sarcopenic indices (i.e., increased SMI, AMI, and walking speed), physical activity, and global WOMAC scores at posttest, compared with the CG receiving RET alone. Second, the EG contained a higher proportion of participants with nonsarcopenia and fewer participants with severe sarcopenia postintervention than did the CG.

### 4.2. Dietary Protein Intake after PS and Muscle Mass Gain

The protein RDA for healthy adults aged over 18 years is 0.8 g/kg/day [85]. In consideration of their higher protein requirement associated with anabolic resistance, the corresponding recommendation for older adults is 1.2 g/kg/day [45,86]. For those engaging in higher physical activity levels [87] or those at high risk of sarcopenia [86,88], the range is 1.2–1.5 g/kg/day. Older adults have been considered at high risk of insufficient dietary protein intake, especially those with acute or chronic conditions such as KOA. According to the results of observational studies, 33.3% to 65.1% of older adults with KOA fail to meet the standard protein RDA [89,90]. Older women with KOA have a mean protein intake of 0.77–0.98 g/kg/day [90,91,92]. In line with previous studies, participants in both groups exhibited a relatively low baseline protein intake of <1.0 g/kg/day (CG, 0.98 g/kg/day; EG, 0.97 g/kg/day), and nearly half of all participants did not meet the protein RDA at baseline. PS successfully enhanced the daily protein intake of the EG to 1.21 g/kg/day, which fulfills the protein intake requirement for individuals undergoing exercise training [87]. By contrast, the protein intake of the CG remained relatively constant (mean = 0.99 g/kg/day). Based on the results of a meta-analysis indicating that an increased daily protein intake of 9.4 g/kg/day attributable to PS accompanies significant increases in the myoprotein synthesis rate in older adults subject to RET [93], the increased daily protein intake of our EG (PS + RET) may result in greater changes in SMI and AMI compared with those in the CG (RET alone).

### 4.3. Effects on Muscle Mass

Evidence regarding the treatment efficacy of PS combined with RET on lean mass gain in older adults has been established in various systematic reviews and meta-analyses [47,94,95,96,97,98,99,100,101]. According to the results of previous meta-analyses, PS augments RET-induced gains in lean body mass [47,94,96,101,102,103] and appendicular lean mass [94,104]) in unhealthy older adults with sarcopenia who are at risk of frailty. In the present study, PS achieved additive effects on changes in SMI and AMI in response to RET, consistent with relevant evidence on individuals with KOA [47,94,102,103,104].

A number of trials had employed PS plus elastic RET for sarcopenic or frail older adults [105,106,107]. Molnar et al. conducted a similar PS dose (33.0 g/session) and exercise protocol (elastic RET, twice weekly for 12 weeks) with the present study, and the authors reported that PS + RET obtained an increase of 1.6 kg of whole-body lean mass more than the RET-alone control [106]. Other authors also conducted the same RET protocol with smaller doses of PS compared with the present study, and the results showed that PS augment effects of RET on appendicular lean mass with greater increases of 0.13–0.37 kg compared to the RET-alone control [105,107]. Our results of muscle mass outcome immediately after PS + RET were in agreement with the previous authors.

Some reasons may explain the findings of muscle mass outcome in this study as follows. First, high protein intake from habitual diet is associated with skeletal muscle mass in older women and men [108,109], especially those with physical disability [110]. In addition, a daily protein intake as high as 1.0–1.2 g/kg/day ensures older adults preserve and gain muscle mass [45]. During RET intervention, our participants in the EG achieved a higher level of protein intake (1.21 g/kg/day) contributed mainly from PS compared to the CG peers (<1.0 g/kg/day). Therefore, participants in the EG appeared to earn greater muscle gains after PS + RET compared to their peers in the CG. Secondly, the increased intake amounts of protein nutrients consumed by our EG participants further resulted in a higher proportion of participants’ calories from protein over study periods, which significantly contributed to counting daily caloric intake, which helped to exceed an adequate intake of calories. Therefore, the EG may achieve significant muscle gains during the study period since inadequate caloric intake negatively impacts muscle mass and its function [111,112,113].

### 4.4. Effects on Muscle Strength Gain

Handgrip strength serves as a clinical indicator of muscle weakness for diagnosing sarcopenia [17,56]. The effects of PS added to exercise on handgrip outcomes in older adults have been widely investigated, with meta-analyses reporting conflicting conclusions—specifically, that they are favorable [94,114,115] or inconclusive [104]. Our results indicate that PS did not augment the effects of RET on MQ (estimated based on handgrip strength). Ikeda et al. combined PS and a home-based RET intervention with elastics in older patients with hip osteoarthritis. Their results for grip strength outcomes between the PS + RET and RET groups concur with ours [116], and increased gains in handgrip strength following PS + RET were noted in both studies. In individuals with lower extremity osteoarthritis, muscle atrophy occurs, especially in muscle groups surrounding the hip or knee joints, in the form of reduced volume or lean mass loss [7,20,117,118]. This close association with muscle weakness further strengthens the relationship between sarcopenia and KOA [18,20,119,120]. However, leg muscle strength appears to be more reliable for determining treatment outcomes following PS + RET than is arm muscle strength in patients with leg osteoarthritis. Therefore, the findings of the present study and of Ikeda et al. may imply that handgrip strength is not an indicator of strength gains, especially for individuals who have osteoarthritis in the hips or knees.

### 4.5. Effects on Function Restoration and Its Associated Factors

In the present study, additional PS was applied for older women with KOA who were undergoing RET, and the results showed that PS + RET was associated with corresponded greater changes in walking speed and WOMWC global scores in response compared to RET-alone control. A number of systematic review studies have investigated the effects of additional PS applied during exercise on physical mobility and global function for sarcopenic or frail elderly people [94,95,115,121,122], and the evidence remains inconclusive. Part of the previous systematic reviews demonstrating that PS augments the effects of exercise on walking speed [95,115], which supports our present results; in contrast, other evidence indicates that additional PS prescribed during exercise intervention does not exert significant effects on changes in walking speed [94,121,122]. The discrepant results between the present study and previous studies can attribute to different populations, and some reasons enable us to explain our findings in the walking-speed outcome as follows. First, due to that previous meta-analyses have indicated that PS plus exercise synergistically increases lean mass along with physical mobility enhancement [95,121,123] and that muscle mass changes in response to PS plus exercise significantly contribute to walking ability restoration in those at high risk of sarcopenia and frailty [115], treatment-induced muscle mass gains in the present study may have contributions in increases in physical mobility, as indicated by performance on the walking speed. However, we did not perform statistical analysis to identify associations between the increases in lean mass and physical mobility levels; therefore, conclusions regarding the effects of lean mass gains on physical performance could not be drawn. Secondly, pain has been identified as one of the determinant factors predicting physical performance in the KOA population [124,125,126], which may explain our findings that greater reductions in WOMAC pain experienced by participants in the EG enabled them to exhibit higher performance in physical mobility at posttest, compared to those in the CG; and such relationship between pain reduction and physical restoration also explain our results of physical activity outcome. Since low skeletal muscle mass is associated with knee pain in patients with KOA [127], targeting muscle mass gains attributable to PS + RET may hold more promise in preventing the progression of physical decline and inactivity in older adults with KOA.

### 4.6. Effects on Sarcopenia Prevention

Low muscle mass is closely associated with osteoarthritis [10,12,14,16,120], which further affects muscle function [128]. In this study, 33.3% and 38.9% participants in the EG and CG, respectively, who had an AMI < 5.7 kg/m^2^ were classified as having sarcopenia or severe sarcopenia at baseline, in line with reports from previous observational studies that the prevalence of low skeletal muscle mass or sarcopenia in older adults with KOA ranges from 15.2% to 57.0% [10,19,129]. After the 12-week intervention, the number of participants with low AMI in the EG (six, 16.7%) was significantly lower than that in the CG (13, 36.1%), and the EG exhibited a greater gain in skeletal muscle mass (AMI) of 0.19 kg/m^2^. Our findings indicate that PS may benefit lean mass gain in older women with KOA undergoing RET; in addition, such alterations in lean mass following PS + RET may influence changes in sarcopenia classifications.

### 4.7. Comparison of Results from Previous Trials Regarding PS for KOA

Protein-based supplementation has been employed in relevant studies to alleviate symptoms and improve function in individuals with KOA [42,43,44,130]. Arjmandi et al. reported that 12 weeks of soy PS (40 g/day) relieved pain and mitigated physical limitations associated with KOA in older women [130]; Zenk et al. [43] and Colker et al. [44] administered milk-based PS to older adults with KOA over 6 weeks. The supplementation significantly improved global WOMAC scores by 30.5% and 15.8%, respectively, from baseline in these two studies. Miller et al. administered herbal leucine to older adults with KOA over 8 weeks, noting that it was significantly more effective in reducing global WOMAC scores (change = 46.5%) than a placebo (change = 25.4%) [42]. In the present study, PS reduced global WOMAC scores (change = 57.9%) more significantly in the EG at posttest than in the CG (change = 45.1%). Our findings are consistent with those from previous studies [42,43,44]. However, compared with previous studies [42,43,44], the present study observed a relatively greater change in global WOMAC scores in the EG. This discrepancy can be explained as follows. First, we administered a PS dose of 31.6 g/d daily, exceeding that in previous trials (2.2–9.0 g/day) [42,43,44]. PS doses as high as approximately 40 g or dietary protein intake exceeding 1.2–1.5 g/kg/day have been recommended for older adults at high risk of sarcopenia [88,131]. In addition, 30–40 g of PS may substantially contribute to the dietary protein intake required for older adults [93,97,132,133,134]. Moreover, for older women, adequate protein intake of >0.8 g/kg/day is associated with higher physical mobility and self-perceived physical function [135]. Taken together, these findings suggest that our EG participants, for whom protein intake increased by 1.21 g/kg/day through PS, may have exhibited greater changes in outcomes of self-perceived physical function than their counterparts in relevant studies. Second, a systematic review and meta-analysis revealed that PS plus exercise exerts a synergistic effect on global function in people with KOA and surpasses PS alone or exercise alone [136]. Accordingly, PS + RET used in the present study may have superior treatment effects on restoring physical function compared with PS alone as employed in previous studies [42,43,44]. Finally, the present intervention period (12 weeks) exceeded that in relevant studies (6–8 weeks) [42,43,44]. Therefore, a longer PS intervention period may achieve greater physical function treatment effects in older adults with frailty and chronic conditions [123].

### 4.8. Limitations

This study had some limitations. First, we enrolled only older women. Based on the sex difference in muscle adaptations to PS and RET [137,138], extrapolating our results to older populations (e.g., older men) with KOA should be conducted with caution. Second, the present study lacked a reference group with no active intervention. Considering the factors such as the Hawthorne effect could not be discounted, fully accounting for the contribution of each intervention to its within-group effects for all outcome measures is challenging [139]. In this study, however, the primary estimates were between-group differences in changes in all outcome measures; the absence of a reference group might not have affected the main conclusions. Third, using a Borg RPE scale that has been designed for aerobic exercise raises concerns regarding the validity and reliability of the self-report exercise effort in the present study, which employed RET. However, the targeted RPE levels (i.e., the ratings ranging from somewhat hard to hard) on the Borg RPE scale are parallel to the OMNI-RES ratings even though the OMNI-RES is optimal to assess the perception of effort for elastic RET. Further studies are warranted to use an OMNI-RES for rating self-report exercise efforts during an elastic RET for older adults. Fourth, we did not control the diet in the CG to match the total caloric intake of the EG during RET intervention since inadequate total caloric intake independently exerts negative impacts on muscle mass and its function [111,112,113]. In addition, the total energy intake and the proportions of macronutrients of habitual diet were not standardized for both groups over the study period. Therefore, the muscle mass outcome after intervention cannot be fully attributed to the effects of PS + RET in the present study. Further studies are warranted to identify the pure efficacy of PS + RET by conducting diet control (including total caloric intake and its proportions of nutrients) during the study period. Finally, the use of pain medication during the study period was not controlled for or assessed, which may have influenced exercise adherence as well as physical mobility performance [140,141]. However, the compliance with attending exercise sessions and the adherence to exercise progressions were equal between the EG and CG in the present study. In addition, patient adherence to home-based strengthening exercise is not associated with changes in pain and strength for such older adults with knee pain [142]. Therefore, the possible use of pain medications appeared to have minor effects on treatment efficacy in this study. Further studies are warranted to identify the effects of pain medication on physical performance following RET.

## 5. Conclusions

In this prospective randomized controlled trial, PS exerted augmentative effects on sarcopenic indices (i.e., increased SMI, AMI, and walking speed), physical activity, and perceived global WOMAC function in older women with KOA through 12 weeks of RET. In addition, PS + RET may mitigate sarcopenia during the intervention period. Based on the results, we recommend that greater emphasis should be placed on PS in combination with RET to prevent sarcopenia and physical inactivity in older adults with KOA, particularly women. The present protocols and findings for PS and elastic RET can facilitate clinical decision-making regarding the optimal rehabilitation strategy for older women with KOA, especially those considered to have sarcopenia.

## Figures and Tables

**Table 1:** Demographic characteristics of the participants.

Variables	CG (*n* = 36)		EG (*n* = 36)		*p* Value
	Mean	SD	Mean	SD	
Sociodemographic					
Age (years)	69.81	7.24	68.64	7.42	0.502 ^a^
Age category					0.635 ^b^
<65 years	6	(16.7)	8	(22.2)	
65–74.9 years	21	(58.3)	17	(47.2)	
≥75 years	9	(25.0)	11	(30.6)	
Marital status, *n* (%)					0.527 ^b^
Married/Partnered	29	(80.6)	31	(86.1)	
Single/Divorced/Widowed	7	(19.4)	5	(13.9)	
Education, *n* (%)					0.630 ^b^
Primary school	11	(30.6)	13	(36.1)	
Secondary school	19	(52.8)	15	(41.7)	
University	6	(16.7)	8	(22.2)	
Living status, *n* (%)					0.394 ^b^
Alone	4	(11.1)	2	(5.6)	
Family/Spouse	32	(88.9)	34	(94.4)	
Smokers, *n* (%)	5	(13.9)	6	(16.7)	0.743 ^b^
Alcohol consumption, *n* (%)	4	(11.1)	6	(16.7)	0.496 ^b^
Number of comorbidities, *n* (%)					0.569 ^b^
1	11	(30.6)	12	(33.3)	
2	11	(30.6)	14	(38.9)	
3	11	(30.6)	6	(16.7)	
≥4	3	(8.3)	4	(11.1)	
Clinical characteristics					
Involved leg, *n* (%)					0.631 ^b^
Right	18	(50.0)	22	(61.1)	
Left	12	(33.3)	9	(25.0)	
Bilateral	6	(16.7)	5	(13.9)	
K–L grade, *n* (%)					0.280 ^b^
I	11	(30.6)	17	(47.2)	
II	19	(52.8)	16	(44.4)	
III	6	(16.7)	3	(8.3)	
Walking aid use, *n* (%)	13	(36.1)	16	(44.4)	0.810 ^b^
CIRS	15.15	4.69	13.74	4.42	0.192 ^a^
Disease duration, years ^c^	9.24	8.25	9.87	8.57	0.751 ^a^
(range)		(0.5–37.8)		(0.4–36.9)	
Slow walking speed, *n* (%) ^d^	32	(88.9)	33	(91.7)	0.691 ^b^
Low muscle strength, *n* (%) ^e^	16	(30.6)	18	(41.7)	0.326 ^b^
Low muscle mass, *n* (%) ^f^	14	(38.9)	12	(33.3)	0.624 ^b^
Sarcopenic grade, *n* (%) ^g^					0.577 ^b^
Nonsarcopenia	1	(2.8)	2	(5.6)	
Presarcopenia	21	(58.3)	22	(61.1)	
Sarcopenia	8	(22.2)	4	(11.1)	
Severe sarcopenia	6	(16.7)	8	(22.2)	
BMI (kg/m^2^)	27.40	3.33	28.10	3.71	0.404 ^a^
Sarcopenia indices					
SMI (kg/m^2^)	14.93	1.91	14.43	1.89	0.267 ^a^
AMI (kg/m^2^)	6.70	1.53	6.67	1.36	0.931 ^a^
Walking speed (m/s)	0.75	0.30	0.72	0.29	0.727 ^a^
MQ (kg/kg)	4.05	1.80	4.02	1.58	0.944 ^a^
PA (MET-hour/week)					
Total	20.90	8.91	19.62	11.06	0.591 ^a^
Vigorous	1.97	1.93	2.07	2.31	0.851 ^a^
Moderate	5.66	3.28	4.79	2.68	0.223 ^a^
Light	13.27	8.91	12.77	10.13	0.830 ^a^
WOMAC					
Global score	59.90	11.19	58.13	10.52	0.493 ^a^
Pain	12.06	2.61	11.22	2.53	0.173 ^a^
Physical difficulty	40.19	9.55	39.00	8.76	0.582 ^a^

^a^ Independent *t*-test; ^b^ Pearson’s chi-squared test; ^c^ Defined as number of years since diagnosis of osteoarthritis; ^d^ Defined as walking speed of <1.0 m/s; ^e^ Defined as handgrip strength of <18 kg; ^f^ Defined as appendicular mass index of <5.7 kg/m^2^; ^g^ Each grade of sarcopenia status was determined based on the conditions of low muscle mass (appendicular mass index of <5.7 kg/m^2^), low muscle strength (handgrip strength of <18 kg), and low physical performance (walking speed of <1.0 m/s); AMI, appendicular mass index; BMI, basal metabolic index; CG, control group; CIRS, Cumulative Illness Rating Scale; EG, experimental group; K–L grade, Kellgren-Lawrence grade for osteoarthritis severity; MQ, muscle quality; PA, physical activity; SD, standard deviation; SMI, skeletal muscle mass index; WOMAC, Western Ontario and McMaster Universities Osteoarthritis Index; PA, physical activity.

**Table 2:** Dietary intake in the experimental and control groups during the study period (excluding supplementation) ^a^.

Variables	Control Group (*n* = 36)		Experimental Group (*n* = 36)	
	Baseline	Posttest	Baseline	Posttest
Energy intake, kcal/day	1567 ± 404	1601 ± 363 *	1499 ± 382	1544 ± 362 *
Protein, g/kg/day	0.98 ± 0.45	0.99 ± 0.44	0.97 ± 0.43	1.02 ± 0.32
Protein, g/day	64.75 ± 28.65	65.21 ± 29.28	63.03 ± 26.86	64.71 ± 28.31
Protein, % ^b^	16.5 ± 5.30	16.5 ± 5.50	16.3 ± 5.1	16.5 ± 5.10
Carbohydrate, g/day	178.7 ± 79.1	195.3 ± 84.2	178.2 ± 73.9	182.2 ± 89.6
Carbohydrate, % ^b^	45.65 ± 4.43	46.82 ± 5.81	47.52 ± 6.02	46.51 ± 5.48
Fat, g/day	62.32 ± 28.35	61.44 ± 17.39	57.46 ± 26.86	58.81 ± 18.94
Fat, % ^b^	35.41 ± 4.79	34.20 ± 3.77	34.50 ± 5.31	35.10 ± 3.92

* Significant time effect, *p* < 0.05. ^a^ Significance level of differences between groups using mixed linear models with age, comorbidity score, disease severity, disease duration, and baseline score as covariates. No significant differences were observed between the experimental and control groups before the intervention. No time × group interaction or main group effects were noted. ^b^ Data denote the percentage contributions of nutrients to daily energy intake.

**Table 3:** Mean changes in primary outcome measures at posttest.

Variables ^a^	CG ^b^		EG ^b^		Difference (EG—CG) ^b^	
	Mean	(95% CI)	Mean	(95% CI)	Mean	(95% CI)
Sarcopenic index						
SMI, kg/m^2^	0.16	(0.04, 0.28) *	0.37	(0.25, 0.49) *	0.21	(0.04, 0.38) *
AMI (kg/m^2^)	0.13	(0.04, 0.21) *	0.33	(0.24, 0.41) *	0.19	(0.07, 0.32) **
Walking speed (m/s)	0.04	(−0.02, 0.10)	0.13	(0.08, 0.18) *	0.09	(0.02, 0.17) *
MQ (kg/kg)	0.48	(0.08, 0.87) *	0.52	(0.12, 0.92) *	0.04	(−0.61, 0.52)
PA (MET-hour/week)						
Total	35.7	(30.1, 41.4) *	65.7	(60.0, 71.4) *	30.0	(21.8, 38.2) ***
Vigorous	11.73	(8.52, 14.95) *	16.71	(13.49, 19.93) *	4.98	(0.36, 9.59) *
Moderate	5.33	(2.99, 7.68) *	8.74	(6.39, 11.09) *	3.41	(0.03, 6.79) *
Light	20.38	(18.61, 22.15) *	38.80	(37.04, 40.57) *	18.42	(15.89, 20.96) ***
WOMAC ^c^						
Global	−26.48	(−27.49, −25.47) *	−34.69	(−35.69, −33.68) *	−8.21	(−9.65, −6.76) ***
Pain	−3.87	(−4.79, −2.95) *	−5.24	(−6.16, −4.33) *	−1.37	(−2.69, −0.05) *
Physical difficulty	−19.21	(−20.01, −18.44) *	−25.71	(−26.51, −24.91) *	−6.49	(−7.64, −5.35) ***

Intention-to-treat analysis: *N* = 72 (control group [CG], *n* = 36; experimental group [EG], *n* = 36). * *p* < 0.05; ** *p* < 0.01; *** *p* < 0.001. ^a^ AMI, appendicular mass index; MQ, muscle quality; PA, physical activity; SMI, skeletal muscle mass index; WOMAC, Western Ontario and McMaster Universities Osteoarthritis Index. ^b^ All analyses were performed using participants’ age, comorbidity score, disease severity disease duration, and baseline score as covariates. ^c^ Negative values denote improvements.

**Table 4:** Effects of protein supplementation plus elastic resistance exercise on sarcopenia.

Classification ^a^	CG (*n* = 36) ^b^					EG (*n* = 36) ^b^					EG *Versus* CG
	Baseline		Posttest		*p* Value ^c^	Baseline		Posttest		*p* Value ^c^	*p* Value ^d^
Nonsarcopenia	1	(2.8)	10	(27.8)	0.004	2	(5.6)	22	(61.1)	<0.001	0.004
Presarcopenia	21	(58.3)	13	(36.1)	0.096	22	(61.1)	8	(22.2)	0.007	0.195
Sarcopenia	8	(22.2)	7	(19.4)	1.000	4	(11.1)	5	(13.9)	1.000	0.527
Severe sarcopenia	6	(16.7)	6	(16.7)	1.000	8	(22.2)	1	(2.8)	0.016	0.047

CG, control group; EG, experimental group. ^a^ Each classification of sarcopenia status was determined based on the conditions of low muscle mass (appendicular mass index < 5.7 kg/m^2^), low muscle strength (handgrip strength < 18 kg), and low physical performance (walking speed < 1.0 m/s). ^b^ All data are presented as *n* (%). ^c^ Analysis using McNemar test to identify effects on changes over time. ^d^ Analysis using Pearson Chi-Square to test for between-group differences at posttest.

## Data Availability

The data presented in this study are available on request from the corresponding author. Public data sharing is not applicable to this article due to ethical considerations and privacy restrictions.

## Supplementary Materials

The following are available online at https://www.mdpi.com/article/10.3390/nu13082487/s1, Table S1: Exercise progression protocol; Table S2: Elastic resistance exercise regime; Table S3: Nutritional composition of the protein supplementation; Table S4: Mean values of primary and secondary outcome measures between the experimental and control groups; and Table S5: Adherence of all patients to the resistance exercise program.
