# Supplementary material for: Effects of Protein-Rich Nutritional Composition Supplementation on Sarcopenia Indices and Physical Activity during Resistance Exercise Training in Older Women with Knee Osteoarthritis
Source: Nutrients. 2021 Jul 21;13(8):2487. doi: 10.3390/nu13082487 (PMC8399515; doi:10.3390/nu13082487)
Supplement: Supplementary file 1 [file nutrients-13-02487-s001.zip › nutrients-1306568-supplementary.pdf]

Table S1. Exercise progression protocol.

Intensity: level of resistance (Theraband color)	Time progression (week)											
	1st	2nd	3rd	4th	5th	6th	7th	8th	9th	10th	11th	12th
	Supervised exercise training		Home-based exercise training									
Yellow	X	X										
Red			X	X								
Green					X	X						
Blue							X	X				
Black									X	X		
Silver											X	X
Exercise Loading												
Repetition	10–20	10–20	10–20	10–20	10–20	10–20	10–20	10–20	10–20	10–20	10–20	10–20
Set	3	3	3	3	3	3	3	3	3	3	3	3
RPE[a]	Moderate–High	Moderate–High	Moderate–High	Moderate–High	Moderate–High	Moderate–High	Moderate–High	Moderate–High	Moderate–High	Moderate–High	Moderate–High	Moderate–High

"X" denoted the intensity of resistance which is determined by Theraband color.
[a]Perception of effort using a 15-point Borg RPE scale. Moderate and high level of RPE correspond to a rating of 13 (somewhat hard) and 15 (hard) respectively, on a Borg RPE scale.
RPE, ratings of perceived exertion.

Table S2. Elastic resistance exercise regime.

Movement	Intensity (Repetition/Set)	Targeted muscle group	Duration (Min)
**A. Warm-up**			
1. Mobility exercise of the neck, upper limbs, and back		Upper quarter flexors and extensors	5
2. Global flexion-extension of the lower limb		Lower quarter flexors and extensors	5
**B. Upper quarter**			
1. Seated chest press	10–20/3	Upper quarter extensors	5–10
2. Seated row	10–20/3	Upper quarter flexors	5–10
3. Seated shoulder press	10–20/3	Shoulder gargle muscle groups	5–10
**C. Lower quarter**			
1. Concentric–eccentric hip circumduction	10–20/3	Hip gargle muscle groups	5–10
2. Leg press	10–20/3	Lower quarter extensors	5–10
3. Leg curl	10–20/3	Lower quarter flexors	5–10
**D. Cool down**			
1. Gentle stretching exercise<br>- Arm stretch<br>- Chest stretch<br>- Core stretch		Upper quarter flexors and extensors	5
2 Gentle stretching exercise<br>- Standing quad stretch<br>- Seated single-leg hamstring stretch<br>- Unilateral knee-to-chest		Lower quarter flexors and extensors	5

Table S3. Nutritional composition of the protein supplementation.

Nutrient	Per serving (24 g)
Energy (Kcal)	85
Protein (g)	14
Fat (g)	0.6
Saturated fat (g)	0.3
Trans fat (g)	0
Carbohydrate (g)	7
Sugar (g)	5
Diet fiber (g)	2
BCAA (g)	4.4
Leucine	2.2
Isoleucine	1.1
Valine	1.1
Glutamine[a]	2.4
Arginine	0.5
Taurine	0.4
Na (mg)	65
K (mg)	80
Ca (mg)	200
P (mg)	120

[a]Including glutamic acid. BCAA, branched-chain amino acid.

Table S4. Mean values of primary and secondary outcome measures between the experimental and control groups.

Variables[a]	Control group (n = 36)[b]		Experimental group (n = 36)[b]	
	Baseline	Posttest	Baseline	Posttest
Sarcopenic indices				
SMI, kg/m^2	14.93 ± 1.91	15.13 ± 1.84	14.43 ± 1.89	14.78 ± 1.95
AMI, kg/m^2	6.70 ± 1.53	6.85 ± 1.35	6.67 ± 1.36	7.04 ± 1.20
Walking speed (m/s)	0.75 ± 0.30	0.77 ± 0.14	0.72 ± 0.29	0.87 ± 0.30
Muscle quality, kg/kg	4.05 ± 1.80	4.49 ± 2.11	4.02 ± 1.58	4.58 ± 1.77
PA (MET-hr/week)				
Total	20.90 ± 8.91	57.97 ± 25.19	19.62 ± 11.06	84.03 ± 39.61
Vigorous	1.97 ± 1.93	13.32 ± 8.89	2.07 ± 2.31	18.94 ± 21.14
Moderate	5.66 ± 3.28	10.71 ± 6.93	4.79 ± 2.68	13.81 ± 7.23
Light	13.27 ± 8.91	33.94 ± 22.79	12.77 ± 10.13	51.27 ± 24.94
WOMAC[c]				
Global (0–100)	59.90 ± 11.26	32.94 ± 8.89	58.13 ± 10.52	24.28 ± 8.03
Pain (0–20)	12.06 ± 2.61	7.81 ± 1.51	11.22 ± 2.53	6.36 ± 3.42
PF (0–68)	40.19 ± 9.55	20.53 ± 3.23	39.00 ± 8.76	13.75 ± 3.33

[a]SMI, skeletal muscle mass index; AMI, appendicular mass index; PA, physical activity; WOMAC, Western Ontario and McMaster Universities Osteoarthritis Index.

[b]Data is presented as mean and standard deviation.

Table S5. Adherence of all patients to the resistance exercise program.

	Resistance level used, n (%)[a]			Exercise volume in each session, mean (SD)						
	Control group	Experimental group	*P* value[b]	Control group			Experimental group			*P* value for total volume[d]
				Set/movement	Repetition/set	Total volume[c]	Set/movement	Repetition/set	Total volume[c]	
Upper quarter										
Yellow	36 (100)	36 (100)		3.22 (0.76)	19.31 (2.12)	61.94 (14.46)	3.17 (0.69)	19.58 (1.84)	62.08 (15.28)	0.969
Red	36 (100)	36 (100)		3.56 (1.16)	19.02 (2.14)	69.03 (24.98)	3.58 (1.13)	18.89 (2.70)	67.92 (24.09)	0.848
Green	36 (100)	36 (100)		3.69 (1.23)	19.17 (2.12)	71.11 (26.27)	3.81 (1.26)	18.75 (2.77)	71.53 (26.13)	0.946
Blue	34 (94.44)	32 (88.89)	0.394	3.68 (1.46)	19.69 (1.25)	71.88 (21.91)	4.24 (1.49)	19.26 (2.18)	80.29 (27.36)	0.171
Black	29 (80.56)	30 (83.33)	0.759	3.46 (1.06)	19.79 (1.82)	67.92 (18.88)	3.80 (1.28)	19.00 (2.62)	70.25 (20.29)	0.697
Silver	26 (72.22)	28 (77.78)	0.586	2.90 (0.31)	17.67 (3.88)	51.33 (12.99)	2.95 (0.24)	18.33 (2.82)	52.29 (10.11)	0.768

[a]Data denotes the number of patients who successfully yielded the indicated resistance level for at least one exercise session.
[b]Pearson Chi-Square test.
[c]Data denotes the mean value of total repetitions acted for each movement and is calculated as set x repetition.
[d]Independent t test.
